# Multiorgan proteomic analysis of infected animal models predict potential host factors for chikungunya virus

**DOI:** 10.1002/mco2.70013

**Published:** 2025-01-03

**Authors:** Dongdong Lin, Cong Tang, Junbin Wang, Yun Yang, Hao Yang, Yanan Zhou, Wenhai Yu, Bai Li, Qing Huang, Haixuan Wang, Ran An, Xiaoming Liang, Yuhuan Yan, Longhai Yuan, Xuena Du, Yuxia Yuan, Yanwen Li, Shuaiyao Lu

**Affiliations:** ^1^ Yunnan Key Laboratory of Cross‐Border Infectious Disease Prevention and New Drug Development Institute of Medical Biology Chinese Academy of Medical Sciences and Peking Union Medical College Kunming China; ^2^ Key Laboratory of Pathogen Infection Prevention and Control (Peking Union Medical College) Ministry of Education Beijing China; ^3^ State Key Laboratory of Respiratory Health and Multimorbidity Beijing China

**Keywords:** animal models, chikungunya virus, host factors, multiorgan proteomics, pathogenesis, targeted therapy

## Abstract

Chikungunya virus (CHIKV) is a mosquito‐borne alphavirus that is primarily known for causing severe joint and muscle symptoms, but its pathological effects have extended beyond these tissues. In this study, we conducted a comprehensive proteomic analysis across various organs in rodent and nonhuman primate models to investigate CHIKV's impact on organs beyond joints and muscles and to identify key host factors involved in its pathogenesis. Our findings reveal significant species‐specific similarities and differences in immune responses and metabolic regulation, with proteins like Interferon‐Stimulated Gene 15 (ISG15) and Retinoic Acid‐Inducible Gene I (RIG‐I) playing crucial roles in the anti‐CHIKV defense. We observed upregulated and downregulated metabolic status in CHIKV‐infected rhesus monkeys and mice, respectively. Additionally, we identified host factors such as S100 Calcium‐Binding Protein A8/A9 (S100A8/A9), Voltage‐Dependent Anion Channel 1/2 (VDAC1/2), Complement Component 3 (C3), Apoptosis‐Inducing Factor Mitochondria‐Associated 1 (AIFM1), Endothelial Cell‐Specific Chemotaxis Regulator (ECSCR), and Kininogen 1 (KNG1) that may contribute to CHIKV‐induced inflammation and hemorrhage. These insights put emphases on the importance of understanding CHIKV's impact on organs beyond joints and muscles, providing potential therapeutic targets and enhancing our understanding of CHIKV pathogenesis. This research underscores the need for appropriate animal models in CHIKV studies and informs the development of targeted therapies to address its systemic effects.

## Graphical abstract

We depicted the multiorgan proteomic landscape in CHIKV‐infected animal models, revealing the potential role of the RLR signaling pathway against CHIKV infection and dysregulated metabolism. Moreover, we predicted potential host factors contributing to organ‐specific inflammation and hemorrhage in rhesus monkeys through bioinformatic and correlation analyses.

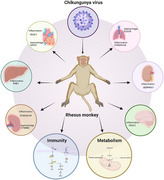

## 1 INTRODUCTION

Chikungunya virus (CHIKV) is an arthropod‐borne alphavirus of the Togaviridae family,^1^, ^2^ which also includes Sindbis Virus, Ross River Virus, and other viruses. CHIKV has an 11.8 kb genome with two open reading frames that encode five structural proteins (C–E3–E2–6K–E1) responsible for forming the viral capsid and envelop protein and four nonstructural proteins (nsP1–4) involved in viral replication.^3^, ^4^ The virus enters host cells by interacting with its envelope proteins and the cellular receptor MXRA8.^5^ CHIKV is primarily transmitted by *Aedes aegypti* and *Aedes albopictus*,^6^ causing symptoms such as fever, skin rash, arthritis, and headache.^7^, ^8^ CHIKV, initially confined to South Africa, has now spread to Asia, Pacific islands, Europe, and the Americans.^9^, ^10^ To date, over 10 million cases of CHIKV infection have been reported in more than 125 countries, with an estimated mortality rate ranging from 0.024 to 0.7%.^11^ Currently, 1.3 billion people living in tropical or subtropical areas are at risk of infection,^12^ and recent outbreaks in temperate regions suggest that climate change could expand CHIKV's reach to highly populated areas, such as the United States, China, and Europe.^10^, ^13^, ^14^, ^15^ Currently, there are no effective approved drugs,^16^ but the first vaccine, Ixchiq(VLA1553) has been approved by the United States Food and Drug Administration, and an mRNA vaccine is under phase 1 trial.^17^ Additionally, mutations have been reported in the envelope gene of the CHIKV, such as E1‐A266V, E1‐K211E, E1‐I317V, and E2‐K211E, which may enhance its adaptability and infectivity.^18^, ^19^ Therefore, it is of essence to understand the pathogenesis of CHIKV to identify potential therapeutical targets.

The host's innate immune response plays a crucial role in defending against CHIKV infection. Recently, studies have highlighted the importance of type I interferon signaling pathway.^20^, ^21^, ^22^ Traditionally, type I interferons (interferon alpha and interferon beta) are produced following the recognition of pathogen‐associated molecular patterns by the pattern recognition receptors (PPRs), leading to the activation of downstream signals.^23^ The primary PPRs^24^ involved include Toll‐like receptors (TLRs), C‐type lectin receptors (CLRs), RIG‐I‐like receptor (RLR) as well as NOD‐like receptor (NLR). Additionally, the cGAS–STING‐mediated signaling pathway can also stimulate type I interferons production. While TLR3.^25^ and STING.^26^ have been shown to inhibit CHIKV replication, the roles of other PRRs in CHIKV defense remain unclear. Furthermore, the intestinal microbiome has been found to inhibit CHIKV replication through a bile acid‐type I IFN signaling axis.^20^

In addition to the innate immune response, the inflammatory response that arises during CHIKV infection is significant, particularly as chronic or persistent polyarthralgia/polyarthritis is a major burden of CHIKV fever.^27^ Some studies have focused on the pathogenesis of arthritic inflammation through joints of CHIKV‐infected animal models or human synovial fibroblast cells. ^28^, ^29^, ^30^, ^31^ However, whether the inflammatory responses trigged by CHIKV infection are systemic remains poorly understood.

Previous studies have explored CHIKV–host interactions, pathogenesis, and pathophysiology.^32^, ^33^, ^34^ However, investigating these aspects through clinical samples is challenging due to ethical constraints. Instead, animal models are a suitable alternative for studying viral infection pathogenesis. Rodent and primate animals are commonly used, each with specific advantages for research.^35^, ^36^ Interestingly, both animal models have been employed in the studies of CHIKV infection. Some comparisons between these two types of animal models have been made,^37^, ^38^, ^39^, ^40^ but the distinction and adaptability between them remain underexplored. Proteomics, a promising strategy for studying viral infection mechanism, allows for comprehensive monitoring of changes, identification of novel targets, elucidation of protein functional networks, and integrative analysis.^41^, ^42^ This approach provides deep insights into virus–host interactions, laying a foundation for understanding infection mechanisms and developing antiviral therapies.

While CHIKV is commonly associated with joint and muscle symptoms, some studies have reported some rare symptoms or diseases.^43^ Also, it has been reported that CHIKV infection can exert a serious impact on organs beyond joints and muscles, such as the heart, brain, kidney, and lung.^44^, ^45^, ^46^ Although some studies have examined CHIKV's impact on these organs, there has been limited comprehensive research on how CHIKV influences systemic organs. Therefore, in our study, we established rodent and nonhuman primate (NHP) animal models, collecting their tissues including heart, liver, spleen, lung, kidney, and brain for proteomic analysis. Our goal is to investigate the broader impact of CHIKV on these organs and to extend the understanding of CHIKV pathogenesis, providing new strategies for the treatment and prevention of CHIKV.

## 2 RESULTS

### 2.1 Distinctions occur in CHIKV‐infected rhesus monkey and mouse models

**FIGURE 1:**
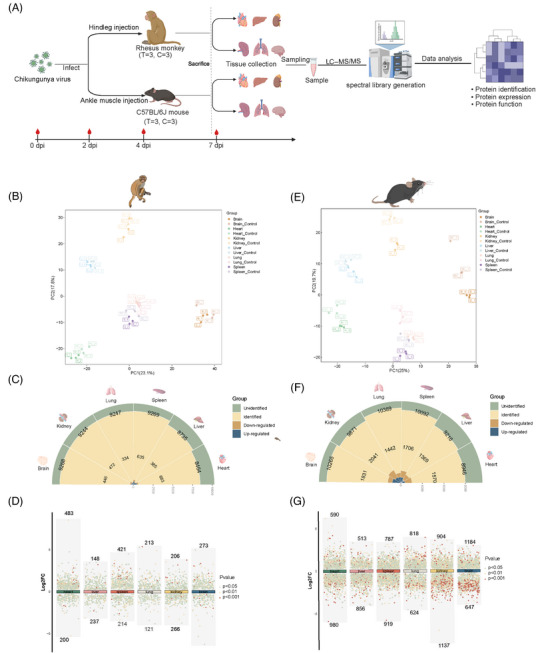
The general proteomic landscape of six organs of CHIKV‐infected rhesus monkeys and mice (A) Experimental design for studying the pathogenesis Chikungunya virus (CHIKV) infection in rhesus monkeys and C57BL/6J mice. (B) The PLS‐DA of proteomic data of six organs from CHIKV‐infected rhesus monkeys. (C) Polarized stack bar plot of quantified and dysregulated proteins in six organs of rhesus monkeys. (D) Sankey plot depicting those DEPs clustered in three or more organs of rhesus monkeys. (E) The PLS‐DA of proteomic data of six organs from CHIKV‐infected mice. (F) Polarized stack bar plot of quantified and dysregulated proteins in six organs of mice. (G) Multidimension volcano plot showing how those identified DEPs were regulated in organs of mice.

In this study, we selected rhesus monkeys (treatment = 3, control = 3) and C57BL/6J mice (treatment = 3, control = 3) as study object to investigate how CHIKV infection impacts organs other than joints and muscles. After infection, we collected blood from two species at different time point and six infected organs including heart, liver, spleen, lung, kidney, brain after they were sacrificed on the seventh day postinfection (dpi) (Figure 1A). Before proceeding with further analysis, we assessed the reliability of our models (Figure S1A–F). RT‐qPCR assays detected CHIKV in the blood of infected rhesus monkeys and mice by the second dpi. However, the virus was no longer detectable in rhesus monkeys by the fourth dpi and was absent in both species by the seventh dpi. Interestingly, CHIKV persisted in the spleen of infected rhesus monkeys, whereas no viral load was detected in the liver of infected mice (Figure S1A,D). Previous studies have suggested that CHIKV can persist in synovial macrophages, leading to tissue damage and a polarized inflammatory response.^47^, ^48^ Similarly, residual SARS‐CoV‐2 has been reported to persist in patients who have recovered from mild Coronavirus Disease 2019 (COVID‐19).^49^ Histopathological findings from hematoxylin and eosin (H&E) staining confirmed that CHIKV infection damages organs beyond joints and muscles (Figure S1B,C,E,F). In infected rhesus monkeys, we observed significant hemorrhage in the heart, spleen, and lungs, lymphocyte infiltration in liver and kidneys, and slight neuronal death and hemorrhage in the brain. In infected mice, significant inflammatory infiltration was observed in the heart, spleen, and lungs, with severe damage to hepatocytes and evidence of neuronal death. Taken together, our findings demonstrate that we successfully established CHIKV‐infected rhesus monkey and mouse models, while also revealing distinct differences between the two models in terms of organ viral load and histopathological outcomes.

### 2.2 Multiorgan proteomic profiling of CHIKV‐infected rhesus monkeys and mice

After obtaining proteomic data from 72 samples, we first performed partial least squares discriminant analysis (PLS‐DA) to determine whether there were similar or distinct organ response patterns to CHIKV infection (Figure 1B,E). We found that protein expression patterns were similar in the spleen and lungs of both species, particularly in rhesus monkeys. However, a distinct separation trend was still observed among the six infected organs in both species. On average, we identified and quantified approximately 8,800 proteins in the rhesus monkey group (heart: 8,464; liver: 8,735; spleen: 9,269; lungs: 9,247; brain: 9,268), which was fewer than the number identified in the mouse group (heart: 8,946; liver: 9,216; spleen: 10,092; lungs: 10,389; kidneys: 9,871; brain: 10,265) (Figure 1C,F). Moreover, the number of differentially expressed proteins (DEPs) was lower in rhesus monkeys than in mice, possibly due to the differing persistence of CHIKV in the organs of the two species. We visualized the distribution of DEPs in the six organs of both species (Figure 1D,G). Interestingly, in mice, the majority of DEPs were downregulated, whereas in rhesus monkeys, most DEPs were upregulated. This species difference may reflect distinct host responses to the same viral infection, and it is also possible that the virus adopts different strategies to promote replication in different hosts. We also investigated whether there were common DEPsbetween the two species (Figure S2A,B). Two proteins, interferon‐induced protein with tetratricopeptide repeats 3 (IFIT3) and ISG15, both involved in the type I interferon signaling pathway,^50^, ^51^ were upregulated in all surveyed organs of both species, suggesting that this pathway was activated in CHIKV‐infected rhesus monkeys and mice. To further investigate the differences between the proteomic profiles of the two animal models, we analyzed the DEPs in the same organs from both species. Except for the liver, we identified numerous common DEPs in rhesus monkeys and mice, though these DEPs displayed either identical or contrasting expression patterns between the two species. We categorized these genes into four distinct groups based on their expression patterns: UP (upregulated in rhesus monkeys), DOWN (downregulated in rhesus monkeys), UP–UP (upregulated in both species in the same organ), and DOWN–DOWN (downregulated in both species) (Figure S3A). Our analysis showed that the heart and kidneys exhibited the highest enrichment of commonly upregulated genes, with 22 and 21 genes identified, respectively. Conversely, the kidneys had the most significant enrichment of commonly downregulated genes, with 37 genes, indicating a shared suppression of specific processes across the two species. The heart and brain had the highest number of genes displaying opposite regulation between the two species, with 92 and 100 genes, respectively. To further explore the conserved or divergent host responses to CHIKV infection between the two species, we conducted functional enrichment analysis (Gene Ontology [GO] and Kyoto Encyclopedia of Genes and Genomes [KEGG]) of the identified genes (Figure S3B). The enrichment analysis of commonly upregulated genes highlighted pathways associated with antiviral responses, the innate immune response, and inflammation, suggesting that both rhesus monkeys and mice activate similar immune‐related pathways to defend against CHIKV infection. In contrast, pathways involved in metabolic processes, such as mitochondrial organization, the tricarboxylic acid cycle, and fatty acid beta‐oxidation, were upregulated in rhesus monkeys but downregulated in mice, indicating a possible divergence in metabolic responses to CHIKV infection between the two species. We then investigated the metabolic proteomic profiles in the two species and conducted further Western blot assays of key metabolic proteins, which confirmed the opposite metabolic responses in rhesus monkeys and mice (Figure S6A,B,D,E). Biochemical tests of metabolic indicators in blood samples from CHIKV‐infected rhesus monkeys also supported these findings (Figure S6C). Taken together, our findings revealed both similarities and differences between the two animal models, emphasizing the complexity of distinct host responses to CHIKV and the importance of carefully selecting an appropriate animal model when investigating various aspects of CHIKV infection.

### 2.3 Proteomic profile of innate immune response identifies significant host factors for distinguishing CHIKV infection

**FIGURE 2:**
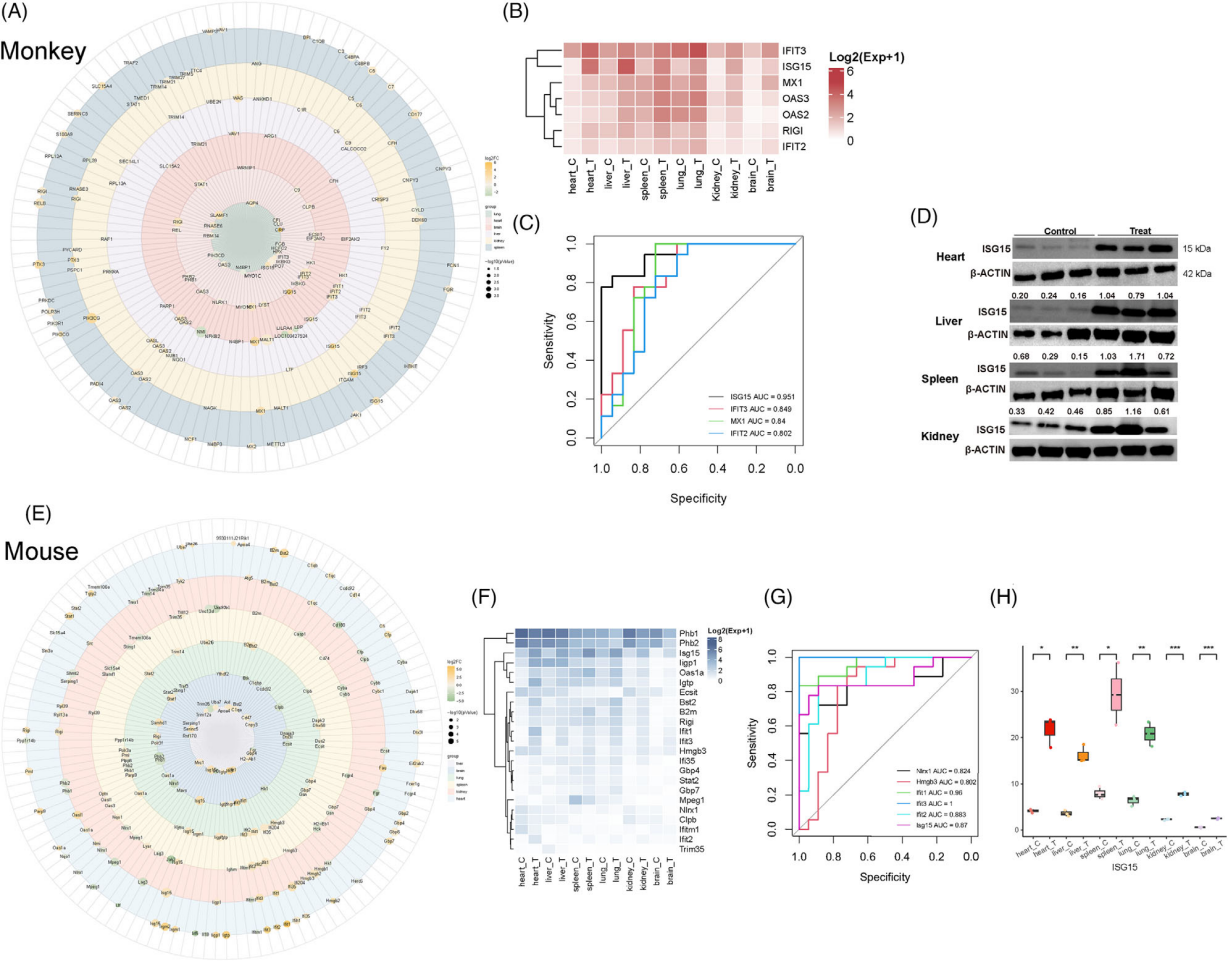
The innate immune profiles in two species following CHIKV infection. (A) Circular bubble matrix of innate immune‐related DEPs in all organs of CHIKV‐infected rhesus monkeys, with six colors representing each organ. (B) Clustered expressive heatmap of DEPs from (A) occurring in three or more organs of rhesus monkeys, with color scale showing the proteomic expression level. (C) ROC curves of those proteins from (B) whose AUC value exceeds 0.8. (D) The western blot results verifying the proteomic expression of 1SG15 in several organs of CHIKV‐infected rhesus monkeys, with number representing the relatively quantified results. (E) Circular bubble matrix of innate immune‐related DEPs in all organs of CHIKV‐infected mice, with six colors representing each organ. (F) Clustered expressive heatmap of DEPs from (E) occurring in three or more organs of mice, with color scale showing the proteomic expression level. (G) ROC curves of those proteins from (F) whose AUC value exceeds 0.8. (H) Box plots showing the proteomic expression of ISG15 in organs of CHIKV‐infected mice with statistical analysis.

To investigate how innate immune responses are triggered in CHIKV‐infected animal models, we analyzed and quantified the differentially expressed innate immune‐related proteins (|fold change| > 1.2 and *p‐* value < 0.05) across six organs in both species (Figure 2A,E). In rhesus monkeys, the liver exhibited the fewest DEPs, while the spleen had the most, suggesting that different organs may employ distinct immune response strategies to CHIKV infection. Similarly, in mice, the liver showed a limited number of innate immune‐related DEPs, whereas the heart, rather than the spleen, had the largest number of DEPs. Functional enrichment analysis of these differentially expressed innate immune proteins revealed both shared and distinct pathways activated in response to the virus in rhesus monkeys (Figure S4A) and mice (Figure S4B). Both species prominently activated key innate immune pathways, including the RLR signaling pathway, TLR signaling pathway, NLR signaling pathway, type I interferon‐mediated signaling pathway, complement activation, and the Nuclear Factor kappa‐light‐chain‐enhancer of activated B cells (NF‐κB) signaling pathway. These pathways are crucial for viral detection, signal transduction, and activation of downstream antiviral responses. Activation of the RLR, TLR, or NLR signaling pathways can trigger the production of type I interferons and other cytokines, driving further antiviral activities and inflammatory responses. However, notable differences were also observed: in rhesus monkeys, pathways involved in the response to cytokines such as Interleukin‐9 (IL‐9), Interleukin‐4 (IL‐4), Interleukin‐1beta (IL‐1β), and Tumor Necrosis Factor (TNF) were upregulated, while in mice, fewer cytokine‐related signaling pathways were identified.

**FIGURE 3:**
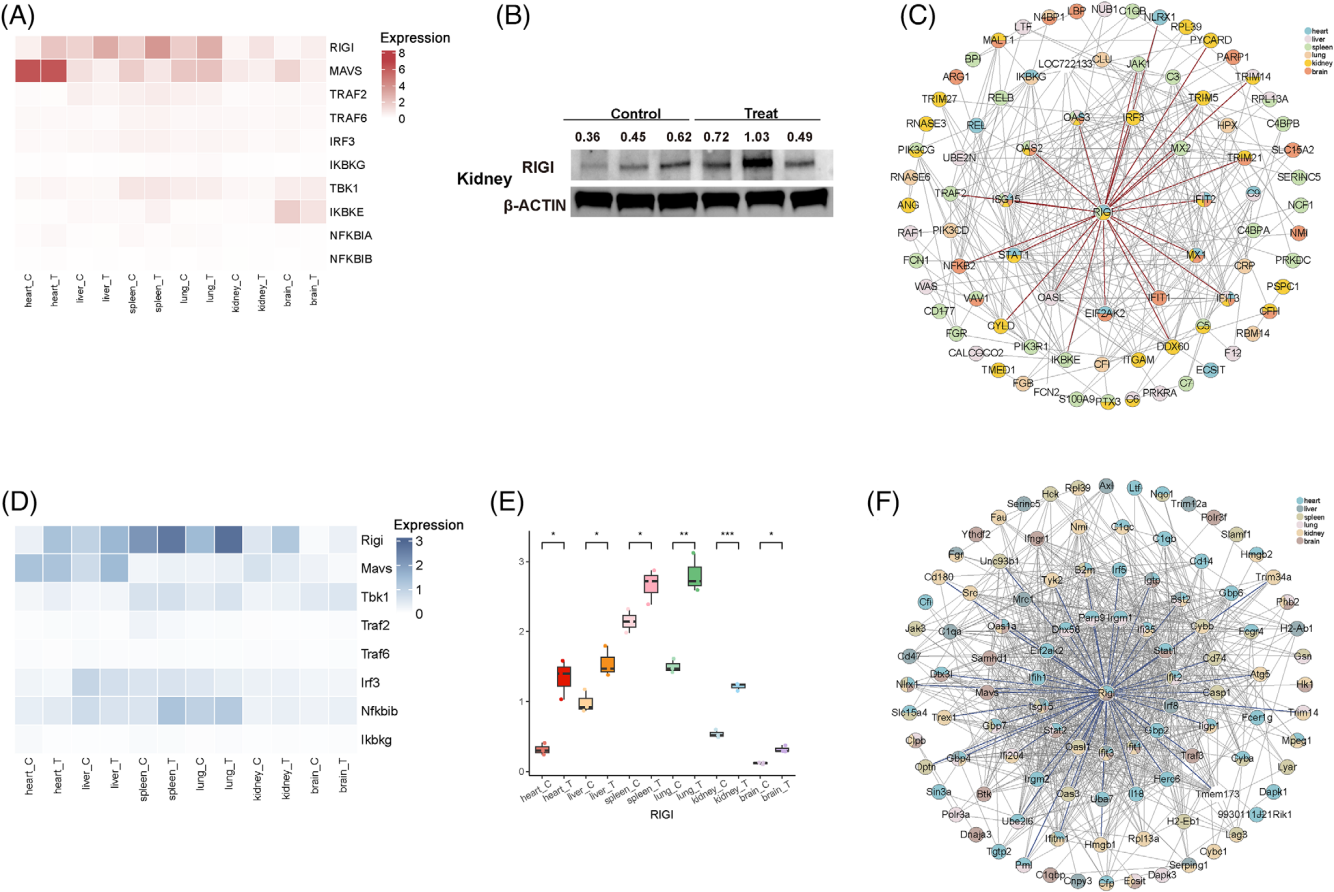
The proteomic profile of RLR signaling pathway in CHIKV‐infected rhesus monkeys and mice. (A) Clustered expressive heatmap of RIG‐I‐like receptor cascade signaling pathway in rhesus monkeys, with color scale showing the proteomic expression level. (B) The western blot results verifying the proteomic expression of RIG‐I in the kidneys of CHIKV‐infected rhesus monkeys, with number representing the relatively quantified results. (C) The protein–protein interaction network of those clustered differentially expressed proteins in CHIKV‐infected rhesus monkeys to identify the antiviral role of RIG‐I by virtue of the string database and application called “Cytoscape,” with color scale representing which organs those proteins are enriched and red line being which proteins are predicted to interact with RIG‐I. (D) Clustered expressive heatmap of RIG‐I‐like receptor cascade signaling pathway in mice, with color scale showing the proteomic expression level. (E) Box plots showing the proteomic expression of Rigi in organs of CHIKV‐infected mice with statistical analysis. (F) The protein–protein interaction network of those clustered differentially expressed proteins in CHIKV‐infected mice to identify the antiviral role of RIG‐I by virtue of the string database and application called “Cytoscape,” with color scale representing which organs those proteins are enriched and red line being which proteins are predicted to interact with Rigi.

We further quantified proteins that were upregulated or downregulated in three or more organs of both animal models (Figure 2B,F). While these proteins showed consistent expression patterns across organs, their expression levels varied. In rhesus monkeys, ISG15, IFIT3, Myxovirus resistance protein 1(MX1), and other proteins involved in interferon signaling and antiviral responses were consistently upregulated across multiple organs, particularly in the liver, spleen, and lungs, indicating a coordinated activation of defense mechanisms and highlighting tissuespecific immune responses. In mice, ISG15 and IFIT3 were also upregulated across multiple organs, mirroring the pattern observed in rhesus monkeys, but the extent of upregulation varied significantly between organs. Notably, the heart, rather than the liver, displayed high expression levels of these proteins in mice, reflecting a species‐specific immune strategy involving multiple organs in antiviral defense. To evaluate the potential of these proteins as diagnostic biomarkers for distinguishing CHIKV‐infected from noninfected states, we performed receiver operating characteristic (ROC) curve analysis on the genes identified in Figure 3B for rhesus monkeys (Figure 2C). The ROC curves for genes such as ISG15, IFIT1, and 2'‐5'‐Oligoadenylate Synthetase 3 (OAS3) demonstrated high Area Under the Curve (AUC) values, with ISG15 achieving an AUC of 0.95, suggesting it could be a highly reliable biomarker for identifying CHIKV infection in this NHP model. Similarly, ROC curve analysis for the genes identified in Figure 3F revealed that ISG15 also displayed high AUC values (AUC = 0.87) in mice (Figure 2G), underscoring its effectiveness in distinguishing between infected and noninfected states in this rodent model. Western blot analysis of ISG15 in rhesus monkey samples (Figure 2D) confirmed its upregulation across multiple organs, consistent with the proteomic data presented in Figure 3B. Similarly, ISG15 was also upregulated in all six organs of mice (Figure 2H). Overall, our study revealed the innate immune profiles in CHIKV‐infected rhesus monkeys and mice, identifying ISG15 as a potential host factor for distinguishing between CHIKV‐infected and healthy states. However, further research is needed to assess its clinical applicability.

### 2.4 RLR signaling pathway, a possible treatment for CHIKV infection

Previous functional enrichment analysis identified several PRR‐related signaling pathways, including TLR, RLR, NLR, and CLR, clustered by DEPs in both CHIKV‐infected animal models. While TLR3 has been reported to inhibit CHIKV replication, the roles of other PRR signaling pathways in this response remains unclear. We specifically focused on the RLR signaling pathway, which detects cytoplasmic viral RNA.^52^ Our analysis revealed that RIG‐I was notably upregulated in multiple organs of rhesus monkeys following CHIKV infection, including the liver, spleen, lungs, and kidneys (Figure 3A). Western blot analysis further confirmed the significant upregulation of RIG‐I at the protein level in the kidneys of rhesus monkeys (Figure 3B). To understand the potential impact of RIG‐I activation, we constructed a protein–protein interaction (PPI) network for all identified innate immune DEPs in rhesus monkeys following CHIKV infection, highlighting the downstream signaling events that might be triggered by RIG‐I activation (Figure 3C). The network analysis suggests that RIG‐I may promote antiviral responses through the activation of the Janus Kinase–Signal Transducer and Activator of Transcription (JAK‐STAT) signaling pathway, ultimately leading to the production of type I interferons and other antiviral molecules. We also investigated the activation of the RLR signaling pathway in CHIKV‐infected mice. As shown in Figure 3D,E, RIG‐I was similarly upregulated in multiple organs, including the heart, liver, and lungs. The PPI network for mice also illustrated that RIG‐I could activate downstream signals such as Mitochondrial Antiviral Signaling Protein (MAVS), facilitating future antiviral activities. Interestingly, more interactions were observed in mice than in rhesus monkeys, suggesting that RIG‐I signaling may influence or be influenced by different signals or pathways in each species.

**FIGURE 4:**
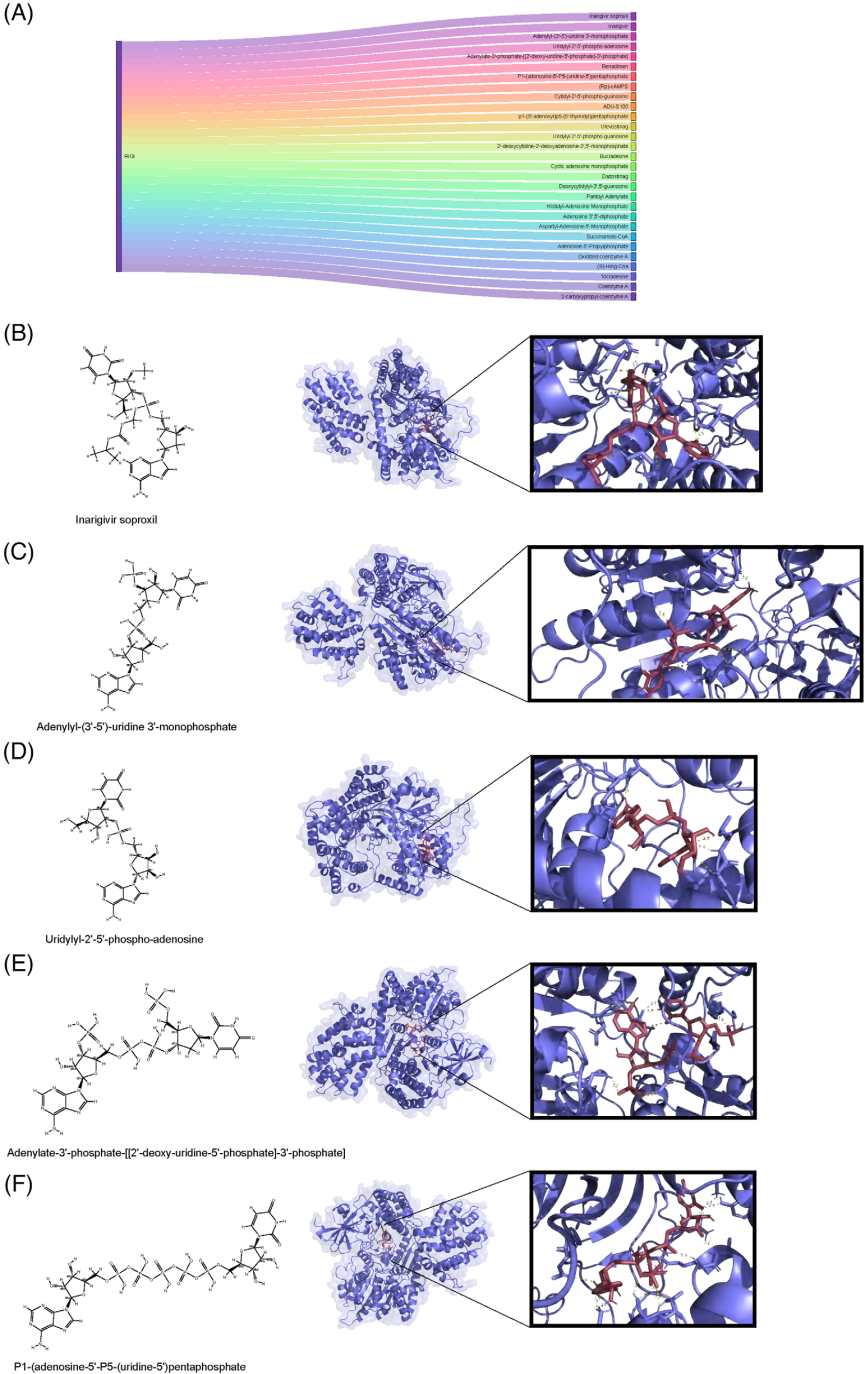
Structure and molecular docking of RIG‐I agonist and its analogues. (A) Sankey plot displaying those possible compounds interacting with RIGI. (B–F) The dimensional forms of inarigivir soproxil and its analogues including inarigivir soproxil, adenylyl‐(3′‐5′)‐uridine3'‐monophosphate, uridylyl‐2′‐5′‐phospho‐adenosine, adenylate‐3′‐phosphate‐[[2′‐deoxy‐uridine‐5′‐phosphate]‐3′‐phosphate], P1‐(adenosine‐5′‐P5‐(uridine‐5′) pentaphosphate, and their molecular docking results with RIG‐I, respectively.

Identifying agonists for RIG‐I could offer a new approach to treating CHIKV infection or inhibiting viral replication. A previous study and clinical trial have reported inarigivir soproxil, a RIG‐I agonist, as effective forHepatitis B Virus (HBV) infection.^53^ Through the DrugBank database, we screened for inarigivir soproxil and its analogues to investigate their potential interactions with RIG‐I using molecular docking simulations (Figure 4A). The similarity scores of the surveyed compounds are listed in Table S1. Furthermore, we selected several compounds with high similarity for molecular docking analysis. Our molecular docking results indicate that these compounds form stable complexes with key RIG‐I binding sites, suggesting their potential as effective RIG‐I agonists. The compounds exhibited multiple hydrogen bonds with RIG‐I, with bond lengths predominantly ranging from 2.0 to 3.5 Å (Figure 4B–F). Short hydrogen bonds (less than 2.7 Å) are generally considered stable and significant,^54^ indicating their high binding affinity with RIG‐I. The docking affinity data further support the potential role of these molecules in interacting with RIG‐I (Table S2). For example, inarigivir soproxil and adenylyl‐(3′‐5′)‐uridine 3′‐monophosphate displayed a binding free energy of −9.4 kcal/mol, indicating robust binding capability. Other compounds, such as uridylyl‐2′‐5′‐phospho‐adenosine (−9.0 kcal/mol) and adenylate‐3′‐phosphate‐[[2′‐deoxy‐uridine‐5′‐phosphate]‐3′‐phosphate] (−8.1 kcal/mol), also demonstrated considerable binding affinity. Overall, our findings suggest that the RLR signaling pathway is activated during CHIKV infection, and RIG‐I agonists like inarigivir soproxil and its structural analogues could play a role in future CHIKV treatment.

### 2.5 Potential organ‐specific and systemic inflammatory targets in organs of rhesus monkeys following CHIKV infection

**FIGURE 5:**
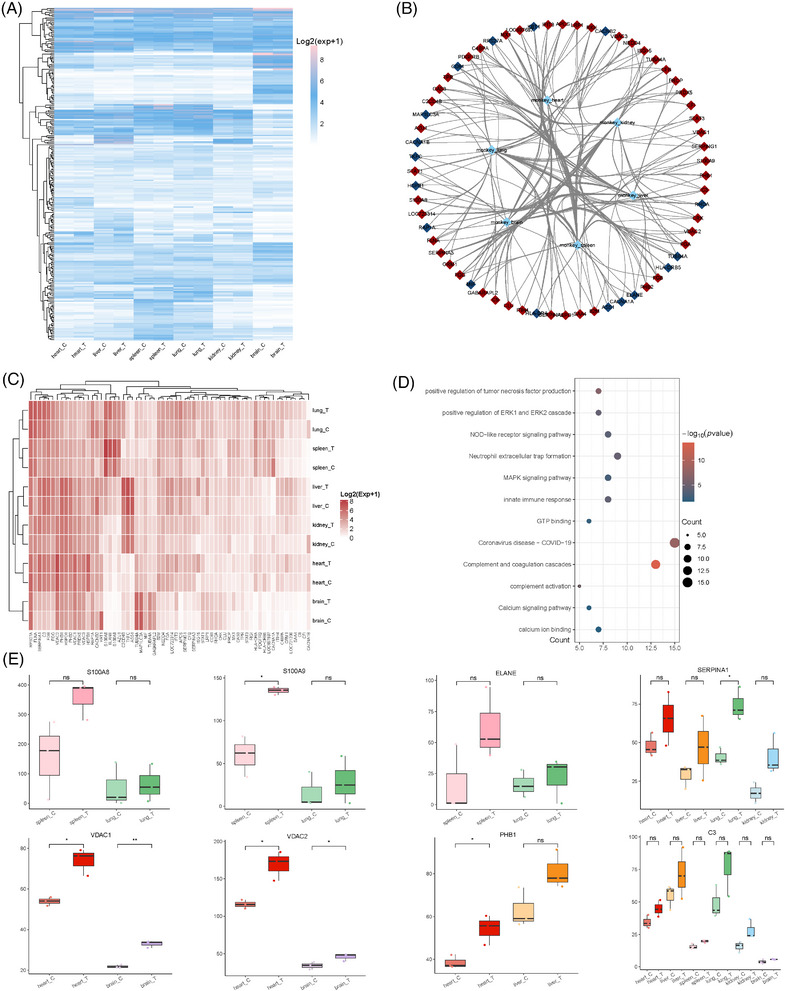
The inflammatory proteomic profile in CHIKV‐infected rhesus monkeys. (A) Heatmap showing the expression of inflammatory response signaling pathway in organs of CHIKV‐infected rhesus monkeys. (B) Network revealing those differentially expressed inflammatory proteins from (A) in each organ (heart, liver, spleen, lung, kidney, brain) of rhesus monkeys. (C) Heatmap showing the expression of filtered proteins in (B) of each organ. (D) Bubble plot showing possible biological function of proteins displayed in (C). (E) Box plot depicting the proteomic expression of identified potential proteins contributing to CHIKV‐induced inflammation with statistical analysis.

To explore the inflammatory responses to CHIKV infection across multiple organs in rhesus monkeys, we analyzed the expression of inflammatory response signaling pathways in all organs (Figure 5A). Our findings revealed organ‐specific expression variations, with the liver, spleen, and lungs showing higher levels of inflammatory gene expression, while the kidneys and brain exhibited lower levels. To focus on proteins involved in systemic inflammation, we filtered this expression matrix to identify proteins enriched in multiple organs and displayed their proteomic expression levels (Figure 5B,C). Interestingly, most of these proteins were upregulated across multiple organs, suggesting that inflammation is activated systemically following CHIKV infection. To determine the signaling pathways involved in the inflammatory response in each organ, we conducted a functional enrichment analysis of these filtered proteins (Figure 5D). The analysis identified several key pathways, including the positive regulation of TNF production, complement and coagulation cascades, neutrophil extracellular trap (NET) formation, Mitogen‐Activated Protein Kinase (MAPK) signaling, and NLR signaling. These results indicate that the inflammatory response to CHIKV involves multiple pathways rather than being confined to a single signaling mechanism.

Finally, we identified potential inflammatory genes that could contribute to the activation of inflammation in various organs (Figure S5E). S100A8, S100A9, and elastase neutrophil expressed (ELANE) were highly expressed in the spleen and lungs of rhesus monkeys. The increased expression of S100A8 and S100A9 suggests a role of neutrophil mediated inflammation in these organs,^55^ as these proteins are key components in the formation of NETs. ELANE, which encodes neutrophil elastase, is known to contribute to tissue damage and inflammation during immune responses.^56^, ^57^ In contrast, inflammation in the heart, liver, and brain may be influenced by mitochondrial dysfunction and energy metabolism disorders. VDAC1 and VDAC2, which participate in mitochondrial function and apoptosis regulation,^58^, ^59^ along with prohibitin 1, involved in mitochondrial dysfunction,^60^ were implicated in these organs. Intriguingly, Alpha‐1‐antitrypsin (SERPINA1) and C3 were upregulated in four organs and all organs, respectively, suggesting that they may be targets for systemic inflammation caused by CHIKV infection. SERPINA1 plays a protective role by inhibiting proteases like neutrophil elastase.^61^ Its deficiency or dysfunction can lead to uncontrolled inflammation,^62^ indicating that SERPINA1 may be a therapeutic target for CHIKV‐evoked inflammation. Complement C3, a critical component of the complement system, showed elevated expression in all organs, particularly in the liver and lungs, indicating that the complement system was activated in response to CHIKV infection in rhesus monkeys. In summary, CHIKV infection triggers systemic inflammation through multiple signaling pathways. The identified potential inflammatory targets may act specifically within certain organs or have systemic effects across multiple organs, warranting further investigation.

### 2.6 Mechanistic insights into CHIKV‐induced hemorrhage across different organs

**FIGURE 6:**
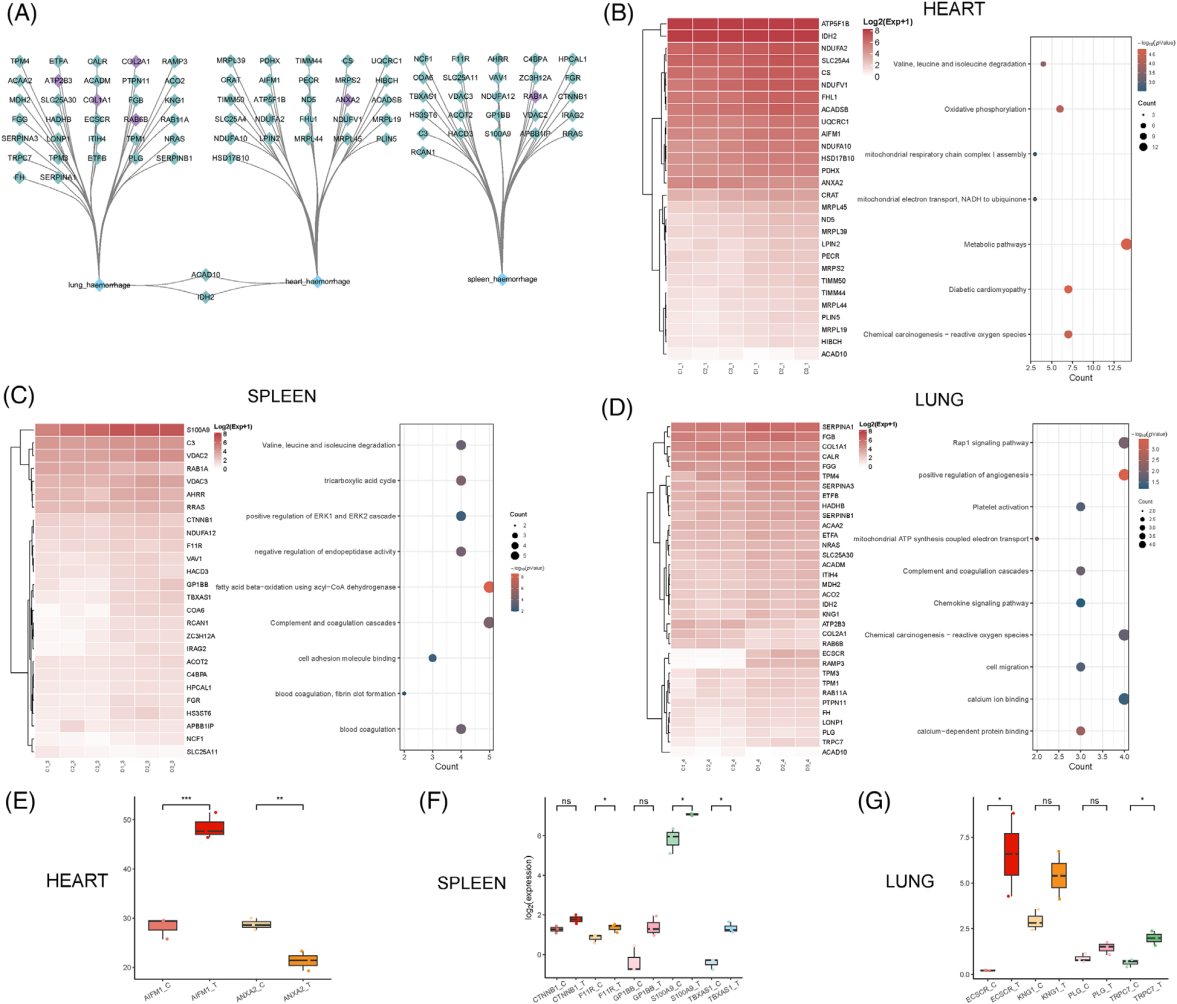
The correlation between hemorrhage and proteomic data in several organs of CHIKV‐infected rhesus monkeys. (A) Correlation network illustrating those highly correlated DEPs in various organs, with blue being positively correlated and purple being negatively correlated. (B–D) Heatmap and bubble plot showing the expression level and potential biological functions of those highly correlated DEPs from (A) in heart, spleen, and lung, respectively. (E) Box plot depicting the proteomic expression of identified potential proteins contributing to CHIKV‐induced histopathological observation of hemorrhage with statistical analysis.

Histopathological characteristics such as vasocongestion/congestion, hemorrhage, and fibrosis have been reported in the organs of patients diagnosed with CHIKV infection.^34^, ^63^ Interestingly, we observed similar histopathological changes in the organs of rhesus monkeys following CHIKV infection, particularly noting hemorrhage in the heart, spleen, and lungs (Figure S1C). To investigate the host factors contributing to this outcome, we conducted a correlation analysis between hemorrhage scores and DEPs in these three organs. This analysis revealed a strong correlation network, with all Spearman coefficients exceeding an absolute value of 0.8 (Figure 6A). We further performed functional enrichment analysis of these DEPs to identify the signaling pathways potentially involved in the observed hemorrhage (Figure 6B–D). In the heart, many DEPs were linked to metabolic pathways, oxidative phosphorylation, and reactive oxygen species (ROS)‐related pathways. In contrast, the DEPs in the spleen and lungs were more associated with immune and blood coagulation pathways, including complement and coagulation cascades, positive regulation of angiogenesis, and platelet activation (Figure 6C,D). These findings suggest that distinct signaling pathways may underlie the hemorrhage observed in different organs.

Our analysis also predicted potential host factors responsible for hemorrhage in these organs (Figure 6E). In the heart, AIFM1 and Annexin A2 (ANXA2) were identified as key proteins likely involved in the hemorrhagic processes. AIFM1 plays a crucial role in apoptosis and maintaining mitochondrial function,^64^, ^65^ while ANXA2 is involved in angiogenesis^66^ and Plasminogen (PLG) activation,^67^ suggesting that its overexpression may contribute to hemorrhage. Interestingly, ANXA2 showed a strong negative correlation with cardiac hemorrhage scores, implying it may be regulated by the host during CHIKV infection. For the spleen, we predicted that Catenin Beta 1(CTNNB1), F11 receptor (F11R, also known as Junctional Adhesion Molecule A [JAM‐A]), Glycoprotein Ib Platelet Subunit Beta (GP1BB), S100A9, and thromboxane A synthase 1 (TBXAS1) could contribute to hemorrhagic processes. CTNNB1, a core component of the Wnt signaling pathway, plays a significant role in cell adhesion,^68^, ^69^ while F11R is essential for maintaining endothelial junction integrity.^70^ GP1BB, a member of the membrane glycoprotein complex Ib‐IX‐V (GPIb‐IX‐V), is essential for primary hemostasis,^71^ and TBXAS1, involved in thromboxane A2 synthesis,^72^ promotes platelet aggregation, indicating that the endothelial or platelet function may influence spleen hemorrhage. In the lungs, ECSCR, KNG1, PLG, and transient receptor potential (TRP) cation channel subfamily C member 7 (TRPC7) were identified as factors that may contribute to pulmonary hemorrhage through various signaling pathways. ECSCR plays a role in angiogenesis and endothelial apoptosis.^73^, ^74^ KNG1 is a precursor of bradykinin,^75^ which is a potent vasodilator involved in the kinin‐kallikrein system regulating blood pressure and vascular permeability.^76^ PLG is a precursor of active serine protease plasmin, which plays a role in fibrinolysis.^77^ TRPC7, a member of the TRP channel family, is involved in calcium ion transport.^78^ These proteins likely impact pulmonary hemorrhage by affecting vascular function in different ways. Overall, our study analyzed host factors in the heart, spleen, and lungs of rhesus monkeys following CHIKV infection, identifying signaling pathways that may contribute to the observed hemorrhage and predicting potential targets for future investigation or treatment of CHIKV‐induced hemorrhage.

## 3 DISCUSSION

In this study, we established and characterized CHIKV infection models in rhesus monkeys and C57BL/6J mice, revealing significant similarities and differences in organ‐specific viral load, histopathology, immunity, and metabolism. Our findings demonstrated that both species exhibited similar innate immune activation across multiple organs, such as the upregulation of type I interferon‐related proteins like ISG15, which emerged as a potential supportive diagnostic target for CHIKV infection. Additionally, the RLR signaling pathway was identified as a promising anti‐CHIKV strategy, with RIG‐I agonists like inarigivir soproxil showing potential for therapeutic application. In rhesus monkeys, we examined the inflammatory responses to CHIKV infection across various organs, identifying several potential host factors, such as S100A8, S100A9, SERPINA1, and C3, which could shed light on the future inflammatory treatments or investigations for CHIKV infection. Moreover, we observed hemorrhagic histopathological changes in the heart, spleen, and lungs of rhesus monkeys, similar to findings in the histopathological analysis of clinical CHIKV cases. Through correlation analysis and bioinformatics, we predicted several host factors, including AIFM1, CTNNB1, and ECSCR, which may contribute to these hemorrhagic manifestations.

The establishment of appropriate animal models is crucial for a comprehensive and precise understanding of viral pathogenesis, as different species may exhibit distinct immune responses and metabolic regulations, potentially affecting the accuracy of research findings. The primary criterion for choosing an animal model is its ability to reflect human disease characteristics as closely as possible. Both rodent and NHP models have been employed in CHIKV pathogenesis research and vaccine assessment.^79^, ^80^, ^81^, ^82^ While mouse models are widely used due to their low cost, easy of management, and the availability of extensive immunological reagents, they exhibit significant limitations in replicating human CHIKV infection,^35^ such as failing to mimic chronic Chikungunya disease, mother‐to‐child transmission, and increased susceptibility in the elderly.^35^ NHP models, particularly rhesus macaques (Macaca mulatta) and cynomolgus macaques (Macaca fascicularis), display clinical symptoms and disease progression similar to those observed in humans, making them valuable for studying aspects of CHIKV pathogenesis that rodent models cannot replicate.^36^ Furthermore, NHP modelsare considered valuable in evaluating the safety and efficacy of vaccines and antibody‐based therapies.^36^, ^82^ the use of NHP models is limited by higher costs, stricter ethical requirements, and other barriers. Despite their respective strengths and limitations, employing and comparing both models remains scientifically valuable, as it allows for a more comprehensive understanding of CHIKV pathogenesis by uncovering species‐specific immune responses and disease progression. Interestingly, it has been reported that a 3D model can be developed for CHIKV infection.^83^, ^84^

Our study revealed significant metabolic differences between rhesus monkeys and mice following CHIKV infection, likely reflecting species‐specific immune response strategies (Figure S6). Previous research has shown that viruses such as Dengue virus, Zika virus, and influenza virus can promote replication by upregulating host metabolic pathways such as glucose and fatty acid metabolism.^85^, ^86^, ^87^, ^88^, ^89^ However, increased energy metabolism, such as oxidative phosphorylation, has also been shown to enhance the production of antiviral effectors like Interferon‐Stimulated Gene 54/56 (ISG54 and ISG56).^90^ Rhesus monkeys may utilize enhanced metabolism to support a more efficient immune defense against CHIKV, while mice may reduce metabolic activity to limit viral replication opportunities. Pathway enrichment analysis of c‐AIMP‐treated CHIKV‐infected cells showed upregulation of metabolic pathways,^26^ and elevated levels of fatty acids and glycerolipids were observed in CHIKV fatalities compared with control groups.^34^ Since metabolism is a dynamic process that can change over the course of infection, the metabolic differences observed between species may be regulated by variations in their immune systems. Further investigation is needed to understand the mechanisms underlying this contrast.

Our findings revealed both shared and distinct innate immune responses to CHIKV infection in the two animal models. Both species activated innate immune pathways, including the RLR, TLR, NLR, type I interferon‐mediated, complement activation, and NF‐κB signaling pathways. Rhesus monkeys exhibited an upregulation of cytokine signaling, including IL‐9, IL‐4, IL‐1β, and TNF, while mice showed fewer activations of cytokine‐related pathways. The distinct innate immune responses between species may be partly due to the single time point of sample collection; therefore, dynamic sampling over time in future studies could provide a more comprehensive understanding of CHIKV infection across different species. Although ISG15 was identified as a strong predictor for distinguishing CHIKV‐infected from noninfected states, its widespread upregulation across various viral infections limits its specificity as a CHIKV‐specific diagnostic marker.^91^, ^92^, ^93^ Consequently, further validation of ISG15 across larger cohorts is needed, and combining ISG15 with other specific biomarkers may enhance diagnostic accuracy.

The RLR signaling pathway emerged as a potential therapeutic target for CHIKV infection, though the expression of MAVS—a crucial adaptor protein in RIG‐I‐mediated antiviral responses—was unexpectedly downregulated in CHIKV‐infected rhesus monkeys while being upregulated in infected mice. Since MAVS is essential for inhibiting CHIKV infection in mice,^94^ understanding the mechanisms behind its downregulation in rhesus monkeys is crucial. Some studies have revealed that RIG‐I and MAVS can be regulated by multiple mechanisms,^52^, ^95^ including posttranslational modifications (PTMs), such as ubiquitylation, phosphorylation, acetylation, interacting proteins (host and viral proteins), and posttranscriptional mechanisms (cellular noncoding RNAs interaction). We investigated the underlying mechanisms for the downregulation of MAVS in rhesus monkeys from the aspect of host protein regulation (Figure S4A). Our analysis in rhesus monkeys revealed that several host factors were likely to explain this situation. NOD‐like receptor family member X1 (NLRX1), a mitochondrial protein that inhibits MAVS by preventing its interaction with RIG‐I,^96^, ^97^ is highly expressed in rhesus monkeys, which may contribute to the observed downregulation of MAVS in this species. Additionally, FK506 Binding Protein 8 (FKBP8)^98^ and Elongation Factor Tu Mitochondrial (TUFM),^99^ both involved in the mitochondrial regulation of MAVS, may also suppress MAVS activity in rhesus monkeys, limiting the downstream signaling of the RLR pathway. In contrast, we observed that these negative regulators of MAVS were downregulated in mice (Figure S4B). Besides, it has been reported that many virus‐encoded proteins can cleave MAVS to inhibit the RLR signaling pathway, such as hepatitis C virus^100^, Seneca Valley virus,^101^ human rhinovirus C,^102^ and coxsackievirus B3.^103^ Whether the underlying mechanism for downregulation of MAVS in rhesus monkeys is due to the host protein regulation or a strategy employed by CHIKV to achieve immune evasion requires further investigations. Despite the downregulation of MAVS in rhesus monkeys, we still found that the downstream signals of MAVS including TANK‐binding Kinase 1 (TBK1), Inhibitor of Nuclear Factor Kappa‐B Kinase Subunit Epsilon(IKBKE) (one of the inhibitors of NF‐κB kinase family members), Interferon Regulatory Factor 3 (IRF3), and TNF‐receptor‐associated factor 6 were upregulated in some organs of rhesus monkeys at low proteomic expression level (Figure 4A). Moreover, we discovered that Tripartite Motif Containing 25 (TRIM25), an E3 ubiquitin ligaseand playing crucial roles in the activation of RIG‐I by promoting its K63‐linked ubiquitination,^104^, ^105^ was upregulated in almost all organs in both species (Figure S4A,B) suggesting a potential host support for RIG‐I activation during CHIKV infection. Similarly, the regulation of 2'‐5'‐Oligoadenylate Synthetase‐Like (OASL), which can enhance RIG‐I signaling by facilitating its oligomerization on viral RNA, may further support the activation of the RLR signaling pathway.^106^ Other host factors involved in the regulation of RIG‐I or MAVS might perform their regulatory roles during CHIKV infection despite their relatively low proteomic expression levels.

We also explored the inflammatory responses to CHIKV infection across multiple organs in rhesus monkeys, identifying several host factors likely involved in CHIKV‐induced inflammation (Figure S5E). Previous research has highlighted the role of cytokines in driving inflammation during CHIKV infection,^34^, ^107^, ^108^, ^109^ our proteomics analysis did not detect elevated cytokine levels. This may be due to several factors: (1) the timing of sample collection which might have missed the peak cytokine production; (2) the limitations of proteomics sensitivity in detecting low‐abundance or rapidly degraded cytokines. We identified host factors such as S100A8, S100A9, and ELANE that were highly expressed in the spleen and lungs. These factors, known to play roles in neutrophilmediated inflammation^110^ and associated with rheumatoid arthritis, may serve as intervention targets for CHIKV‐induced arthritis.^111^, ^112^, ^113^ Hemorrhagic manifestations, observed in clinical CHIKV cases.,^34^, ^46^ were also found in some organs of CHIKV‐infected rhesus monkeys (Figure S1C). Therefore, we performed Spearman correlation analysis (with a threshold of 0.8) to identify the correlation between hemorrhage scores and DEPs in the heart, spleen and lungs of CHIKV‐infected rhesus monkeys. The Spearman correlation coefficient is suitable for non‐normally distributed continuous data.^114^, ^115^ The functional enrichment analysis of different organs revealed that hemorrhage in the heart, spleen, and lungs may be regulated by distinct signaling pathways. Heart hemorrhage was closely associated with metabolic pathways, oxidative phosphorylation, and ROS‐related pathways, with the AIFM1 and ANXA2 being predicted to be involved in heart hemorrhage. In contrast, spleen and lung hemorrhage were associated with immune and blood coagulation pathways (such as complement and coagulation cascades, platelet activation, and angiogenesis), while we identified CTNNB1, F11R, ECSCR, KNG1, and other proteins, which may exert an impact on spleen and lung hemorrhage by regulating vascular function.

Our study has several limitations. First, the single sample collection time may have missed dynamic immune responses at other stages of CHIKV infection. Second, the contrasting metabolic responses in CHIKV‐infected rhesus monkeys and mice were not fully elucidated. Third, the lack of functional validation experiments for the identified host factors means their roles remain predictive. Finally, our study did not integrate multiomics data, which could provide a more comprehensive understanding of CHIKV pathogenesis. Therefore, future studies should address these limitations by adding dynamic proteomic and metabolic analyses, functional validation of key factors in both in vivo and in vitro models, and multiomics integration.

In conclusion, our multiorgan and multispecies proteomic analysis provides valuable insights into the pathogenesis of CHIKV infection. By identifying potentially key host factors associated with innate immune responses, inflammation, and organ hemorrhage across different organs, our research offers both crucial clues and mechanistic insights that may deepen our understanding of CHIKV infection characteristics. These findings not only enhance our knowledge of how CHIKV impacts various organs beyond joints and muscles, but also predict host factors across different organs as potential therapeutic targets that could be explored in future studies. Overall, our study lays a strong foundation for further research into CHIKV infection.

## 4 MATERIALS AND METHODS

### 4.1 Animals, ethics, and biosafety statement

Rhesus monkeys and mice were obtained from the Institute of Medical Biology, Chinese Academy of Medical Sciences (license number: SCXK(DIAN)K2020‐0005, SYXK(DIAN)2022‐0006). All approved animal experiments were conducted with the support of the Institutional Animal Care and Use Committee of the Institute of Medical Biology, Chinese Academy of Medical Science(ethics number: DWSP202110006), and strictly followed the guidelines in the Animal Biosafety Level 3 (ABSL‐3) facility of the Kunming National Primate Research Centre of High‐level Bio‐safety, Yunnan, China.

### 4.2 Rhesus monkey and mouse systematic organs preparation

All NHPs chosen in this study were derived from 7 to 14 years old rhesus monkeys (male and female) and are completely healthy without any infections, arthritis, and other diseases. All rodents chosen in this study were derived from 6 to 8 weeks old C57BL/6J (male and female) and are completely healthy without any infections, arthritis, and other diseases. The virus strain used for this study is imported from Malaysia to China, with its gene bank accession number being MH670649 from the National Center for Biotechnology Information (NCBI) (https://www.ncbi.nlm.nih.gov/nuccore/MH670649.1/). The dose of CHIKV used for this research was 2 × 10^7^ PFU. After being transferred to the laboratory, all rhesus monkeys underwent a period of adaptation for 1–2 days before receiving an injection into their hind legs, with a volume of 333 µL per leg and a total volume of 666 µL, and then the animals were euthanized at 7 dpi after the challenge and dissected for sampling. Similarly, all C57BL/6J mice were transferred to the laboratory and allowed to adapt for 1 day before being injected near the ankle muscle using a microsyringe. Each leg received an injection volume of 50 µL, for a total volume of 100 µL. The animals were then euthanized at 7 dpi after the challenge and dissected for sampling. Organ collection occured at 0 and 7 dpi, with the former serving as a mock group and the latter as a treated group. At each time point of organ sampling, approximately 150  mg of each organ was collected and then they will be put with Phosphate‐Buffered Saline (PBS) for further centrifugation. Afterward, all sediments were heated at 56°C for half an hour.

### 4.3 RT‐qPCR for the detection of viral load

For blood samples, they were inactivated by mixing with Trizol in the ratio of 1:3 (50 µL:150 µL). For tissue samples, approximately 150 mg was mixed with 800 µL Trizol, followed by inactivation and thorough homogenization. After these preparations, 200 µL of Trizol‐sample mixture was extracted using the UPure Virus RNA Plus Kit (Cat#M2006P‐A96; Biokeystone, China), and the extracted nucleic acids were used for RT‐qPCR. RT‐qPCR was performed using TaqMan Fast Virus 1‐Step Master Mix (Catalog Numbers 4446P‐A96; Biokeystone) (Cat#4444432; Thermo Fisher Scientific, USA) for detection, with the CFX384 Touch Real‐Time PCR Detection System (Bio‐Rad, USA). Primers and probes synthesized to target viral structural proteins were as follows:

CHIKV‐F: 5′‐AAGCTCCGCGTCCTTTACCAAG‐3′;

CHIKV‐R: 5′‐ CCAAATTGTCCTGGTCTTCCT‐3′

CHIKV‐Probe: 5′‐FAM‐CCAATGTCTTCAGCCTGGACACCTTT‐TAMRA‐3′. Plasmids containing the target were used as standard samples after gradient dilution. One‐step RT‐PCR was performed under the following conditions: 25°C for 2 min, 50°C for 15 min, 95°C for 2 min, followed by 40 cycles of 5s at 95℃ and 31 s at 60°C.

### 4.4 Protein extraction, digestion, and desalination

Three hundred microliters of 8 M  urea and protease inhibitor (10% of the lysate volume) were added to the sample. And then the supernatant was then collected after centrifugation (14,100×*g*, 20 min), and 100 µg of extracted proteins was incubated with 200 mM Dithiothreitol (DTT) at 37°C for 1 h for protein reduction. The sample was then diluted fourfold with 25 mM ammonium bicarbonate (ABC) buffer, followed by trypsin digestion (1:50, trypsin) at 37°C overnight.

The next day, 50 µL of 0.1% formic acid (FA) was added to stop the digestion. The C18 column was washed with 100 µL of 100% Acetonitrile (ACN), followed by 100 µL of 0.1% FA, centrifuging at 140x*g* for 3  minutes after each step. The EP tube was replaced, the sample was loaded onto the column, and centrifugation was repeated at 1200 rpm for 3 minutes. The column was washed twice with 100 µL of 0.1% FA, followed by one wash with 100 µL of pH 10 water. After replacing the EP tube, elution was performed with 70% ACN. The eluates were combined, lyophilized, and stored at −80°C until use.

### 4.5 H&E staining

Histological analysis was conducted using H&E staining, a classical histopathological technique widely employed to visualize cellular structures and tissue morphology. Hematoxylin selectively stains nuclei in a blue hue, while Eosin imparts a pink color to cytoplasmic components, enabling comprehensive and detailed tissue examination.

### 4.6 Biochemical indicators (TG, GLU, LP) analysis

All reagents used for detecting biochemical indicators are purchased from Shanghai Kehua Bio‐engineering Co., Ltd., and the accompanying biochemical analysis equipment (ZY‐1280) was also purchased from the same company. The blood was collected from the small saphenous vein of the hind limb at 0 and 7 dpi respectively, and the serum was centrifuged at 2,851x*g* for 10 minutes, which was then stored in the refrigerator at −80°C for future use. After serum collectionwas collected at all the time points, the ZY‐1280 automatic biochemistry analyzer and its matching reagents produced by Shanghai Ke hua Bio‐engineering Co., Ltd., were used for analysis. The reagent used were GLU (Kehua, Cat#30812020401), TC (Kehua, Cat#30812070912), and TG (Kehua, Cat#30812071015).

### 4.7 Antibodies

Antibodies against ISG15 (Proteintech; Cat#15981‐1‐AP, Dilution 1:2000), Hydroxyacyl‐CoA Dehydrogenase Trifunctional Multienzyme Complex Subunit Alpha (HADHA) (Proteintech; Cat#10758‐1‐AP, Dilution 1:2000), Dihydrolipoamide S‐Acetyltransferase (DLAT) (Proteintech; Cat#13426‐1‐AP, Dilution 1:2000), Carnitine Palmitoyltransferase 2 (CPT2) (Proteintech; Cat#26555‐1‐AP, Dilution 1:2000), Beta‐Actin (Proteintech; Cat#HRP‐66009, Dilution 1:5000), and RIGI (Proteintech; Cat# 20566‐1‐AP, Dilution 1:1000) were purchased from the indicated manufacturers. Secondary antibodies, including HRP goat anti‐rabbit IgG (ImmunoWay; Cat#RS0002, Dilution 1:10,000), were purchased from the indicated manufacturers.

### 4.8 Western blot analysis

Protein samples from infected tissues were subjected to SDS‐PAGE and transferred to polyvinylidene difluoride membranes (Bio‐Rad; Cat#1620177). After blocking with blocking buffer (Bio‐Rad; Cat#12010020), the membranes were incubated with primary antibodies overnight at 4°C, followed by incubation with the corresponding secondary antibodies for 1 hour at room temperature. Protein signals were detected by ChemiDoc MP lmaging System (Bio‐Rad; Cat#12003154).

### 4.9 LC–MS/MS analysis

For spectral library generation, a high‐pH reversed‐phase chromatography was used for sample fractionation and data‐dependent acquisition (DDA) mode for analysis. A Q Exactive HF‐X mass spectrometer (Thermo Fisher Scientific) coupled to an EASY nLC 1200 system was used, with an 80‐minute run time and the column temperature maintained at 60 ℃. For data‐independent acquisition (DIA), the method included an MS1 scan (350–1500 *m*/*z*, resolution 60,000, maximum injection time 50 ms, AGC target 3E6) followed by 42 MS2 segments with isolation windows ranging from 14 to 312 *m*/*z* (resolution 30,000, maximum injection time 54 ms, AGC target 1E6). Stepped collision energies were set at 25, 27.5, and 30, with the default MS2 charge state at 3.

### 4.10 Proteomic data analysis

Mass spectrometry data processing involved generating a DDA library from fractionated pools (DDA MS data, six fractions) and a direct‐DIA library from single‐shot samples (DIA MS data), which were then merged into a hybrid library using Spectronaut software (Biognosys; version 15.7.220308.50606). This hybrid library was used for protein identification and quantification in single‐shot samples, with all searches performed against the *Macaca mulatta* Uniprot proteome database (44,389 sequences, downloaded on 2023‐07‐24). Fixed modifications included carbamidomethylation, while variable modifications were acetylation of protein N‐termini and oxidation of methionines. Trypsin/P was set as the cleavage enzyme, allowing two missed cleavages, and peptides of 7–52 amino acids were analyzed. Protein intensities were normalized using Spectronaut's “Local Normalization” algorithm. For the spectral library, a minimum of three fragments per peptide and a maximum of six were selected, with a 1% FDR for both protein and precursor. Protein quantities were reported only if they passed the *Q*‐value sparse filter.

### 4.11 Bioinformatic analysis

The bar plot, pie plot, bubble plot, and volcano plot were visualized with ggplot 2 (a package of R package: https://www.r‐project.org/) and GraphPad Prism 8.0. Upset diagram is generated by UpsetR (a package of R language). GO and KEGG enrichment analysis of DEPs in the category of biological process and pathway, respectively, is conducted with DAVID (https://david.ncifcrf.gov/) and visualized with ggplot2. Heatmaps of DEPs expression are generated by Pheatmap and Circlize package. The PLS‐DA analysis and the chord diagram are achieved by the online website called “Biodeep” (https://www.biodeep.cn/home). The Search Tool for the Retrieval of Interacting Genes/Proteins(STRING) database (https://cn.string‐db.org/) is used for establishing PPI networks of the DEPs, while network visualization is achieved by the Cytoscape 3.8.0 (https://cytoscape.org/) application on the basis of network information from STRING mentioned above.

### 4.12 Molecular docking analysis

Protein structures in PDB format were downloaded from the Protein Data Bank (PDB), and small molecule ligands in SDF format were obtained from the PubChem database. The protein structures were then optimized in PyMOL (v3.0.3) by removing solvent molecules and other irrelevant components, while the small molecule ligands were subjected to energy minimization in Chem3D Pro (v14.0) to achieve the lowest energy conformation, resulting in MOL2 format files. Subsequently, the protein files were hydrogenated, and the optimized ligands were imported into AutoDock Tools (v1.5.7). The binding site on the protein was identified, and both protein and ligand files were exported in PDBQT format for molecular docking. AutoDock Vina (v1.1.2) was then used to perform the docking studies, with the lowest binding energy conformation representing the binding affinity. Finally, the conformation with the lowest activation energy was visualized in PyMOL, focusing on hydrogen bond interactions, and a molecular docking image was generated for analysis.

### 4.13 Statistical analysis

All statistical analyses are performed using statistics‐based packages of R language. *p *< 0.05, *p *< 0.01, and *p *< 0.001 are considered to indicate different significant levels.

## AUTHOR CONTRIBUTIONS

Research design: D. L., C. T., and S. L. Infect Rhesus monkeys and mice, sample collection: C. T., J. W., H. Y., and Y. Z. Sample sequencing outsource: Y. Y., W. Y., B. L., Q. H., and R. A. Histopathological work: C. T. and S. L. Proteomic data analysis and figure drawing: D. L. Data collection: X. L. and Y. Y. Figure beautification, data collection: L. Y., X. D., Y. Y., and Y. L. Paper writing: D. L. Writing review: S. L. All authors have read and approved the final manuscript.

## CONFLICT OF INTEREST STATEMENT

The authors declare that they have no conflict of interest.

## ETHICS STATEMENT

The experimental operation complied with the 3R principles of animal ethics and was approved by the Animal Ethics Committee of the Institute of Medical Biology, Chinese Academy of Medical Sciences (DWSP202101001) and performed in strict accordance with the guidelines for the National Care and Use of Animals approved by the National Animal Research Authority (PR China).

## Supporting information



Supporting Information

## Data Availability

The high‐throughput proteomics data generated in this study have been deposited in the PRIDE database (https://www.ebi.ac.uk/pride/) and are publicly available with the accession number (PXD048726).
