# Supplementary material for: Multiorgan proteomic analysis of infected animal models predict potential host factors for chikungunya virus
Source: MedComm (2020). 2025 Jan 3;6(1):e70013. doi: 10.1002/mco2.70013 (PMC11702418; doi:10.1002/mco2.70013)
Supplement: Supplementary file 1 — Supporting Information [file MCO2-6-e70013-s001.pdf]

Supplemental Materials

Multi-Organ proteomic analysis of infected animal models predict potential host factors for chikungunya virus

Dongdong Lin[1#], Cong Tang[1#], Junbin Wang[1#], Yun Yang[1], Hao Yang[1], Yanan Zhou[1], Wenhai Yu[1], Bai Li[1], Qing Huang[1], Haixuan Wang[1], Ran An[1], Xiaoming Liang[1], Yuhuan Yan[1], Longhai Yuan[1], Xuena Du[1], Yuxia Yuan[1], Yanwen Li[1], Shuaiyao Lu[1,2,3,4*]

Author affiliations: 1. Institute of Medical Biology, Chinese Academy of Medical Sciences and Peking Union Medical College, Kunming, China; 2. Key Laboratory of Pathogen Infection Prevention and Control (Peking Union Medical College), Ministry of Education, Beijing, China; 3. State Key Laboratory of Respiratory Health and Multimorbidity, Beijing, China; 4. Yunnan Provincial Key Laboratory of Vector-borne Diseases Control and Research, Kunming, China

# These authors contribute equally.

* The correspondence author:

Shuaiyao Lu, lushuaiyao-km@163.com

This PDF file includes:

Figure S1-S6 and Table S1 and S2.

Figure S1

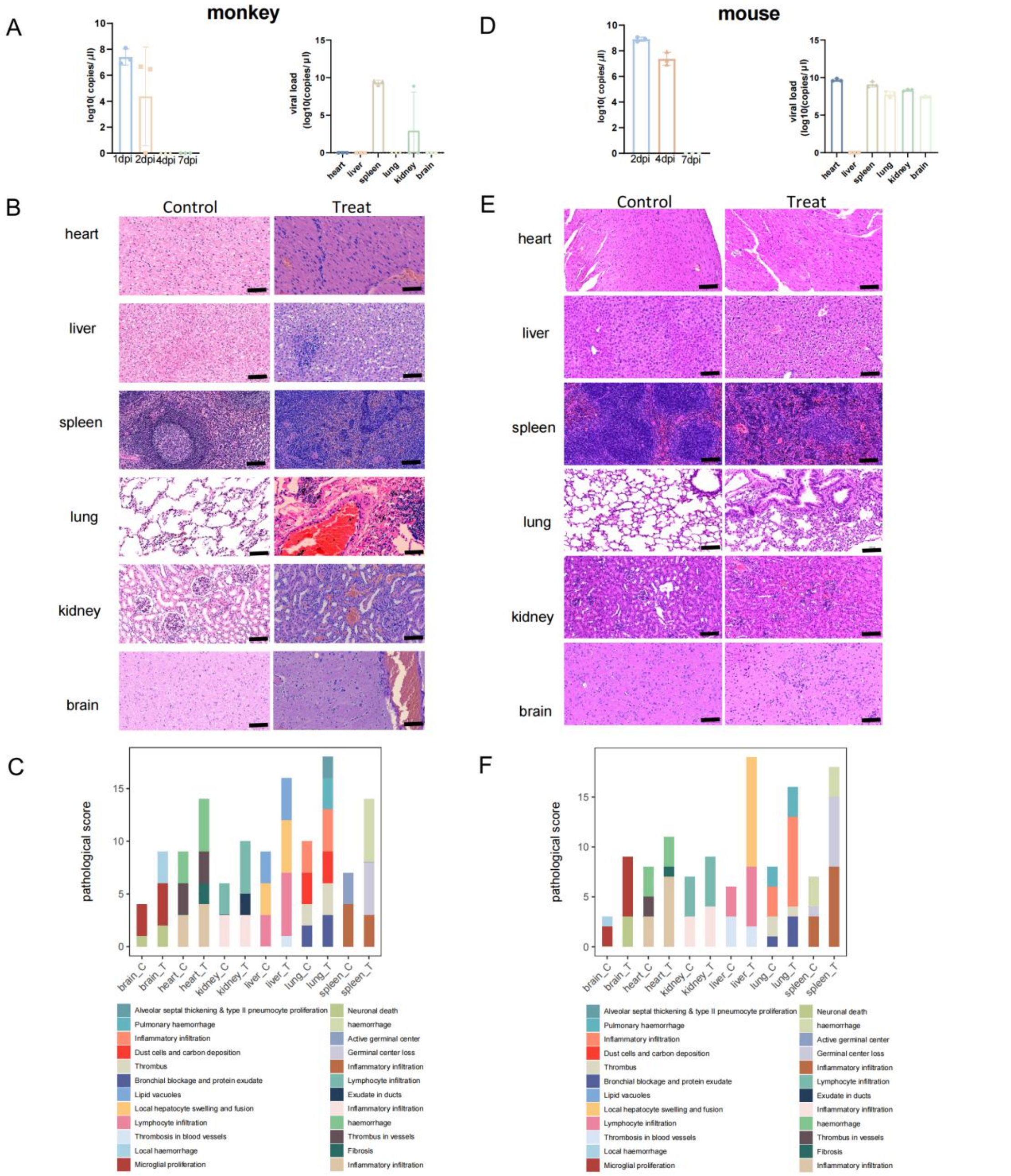

Fig.S1 The assessment of CHIKV-infected rhesus monkey and mouse models. (A) Bar plot showing the expression of CHIKV in blood at different challenging time and in organs on the sacrificed day of rhesus monkeys. (B) Representative H&E-stained results of each surveyed organ (Heart, Liver, Spleen, Lung, Kidney, Brain) in rhesus monkeys. (C) The relevant pathological scores based on their H&E results of rhesus monkeys, with colour representing different assessment contents for organs. (D) Bar plot showing the expression of CHIKV in blood at different challenging time and in organs on the sacrificed day of mice. (E) Representative H&E-stained results of each surveyed organ (Heart, Liver, Spleen, Lung, Kidney, Brain) in mice. (F) The relevant pathological scores based on their H&E results of mice, with colour representing different assessment contents for organs.

Figure S2

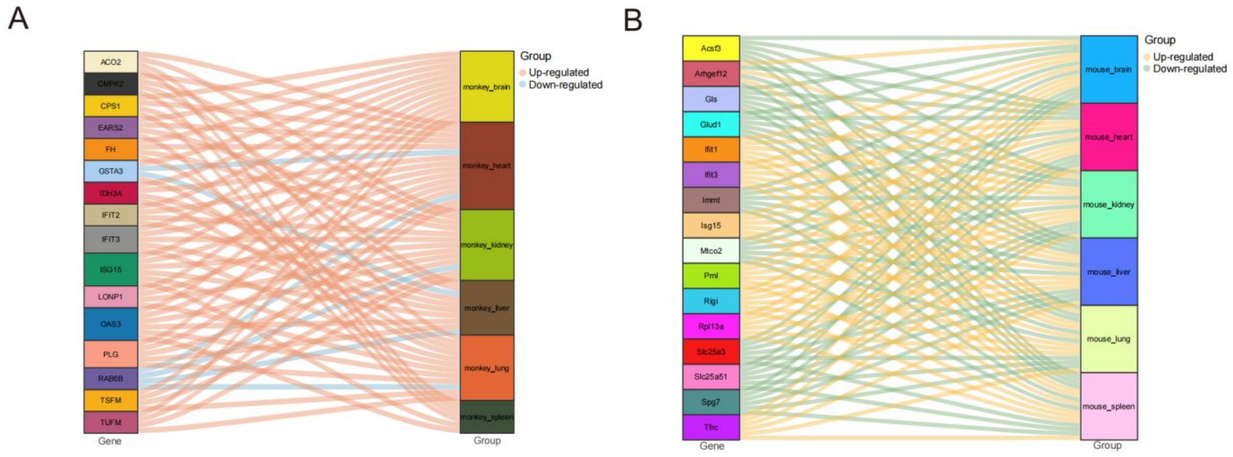

Fig.S2 Common DEPs in three or more organs of CHIKV-infected rhesus monkey and mouse models. (A) multi-dimension volcano plot showing how those identified DEPs were regulated in organs of rhesus monkeys. (B) Sankey plot depicting those DEPs clustered in three or more organs of mice.

Figure S3

A

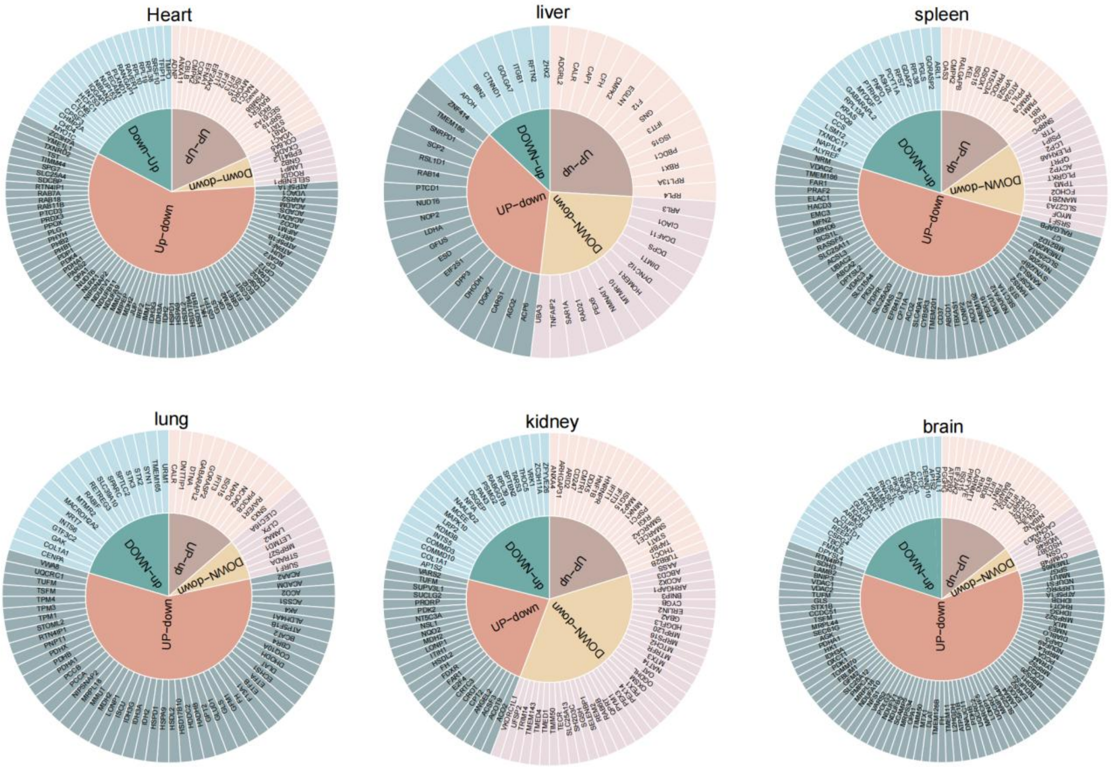

B

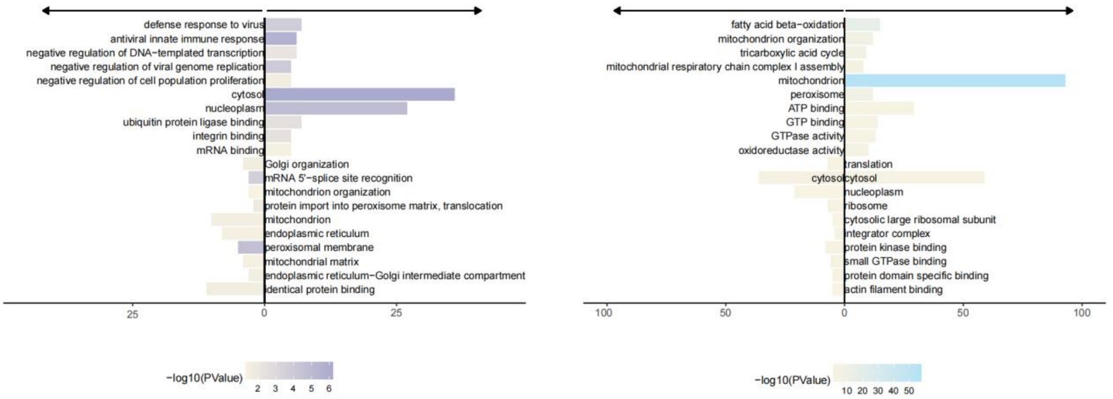

Fig.S3 The DEPs consistence and differences of the proteomic of CHIKV-infected rhesus monkeys and mice. (A) circular plots showing those commonly or contrarily regulated DEPs in the same organ of CHIKV-infected rhesus monkeys and mice. "UP" and "DOWN" represent those upregulated and downregulated DEPs in rhesus monkeys respectively, while "Up" and "Down" represent those upregulated and downregulated DEPs in mice respectively. (B) Bar plots showing the functional enrichment result of those commonly or contrarily regulated DEPs in two species, with the left panel representing the enrichment results of commonly regulated DEPs in two species, while the right panel representing the enrichment results of contrarily regulated DEPs in two species.

Figure S4

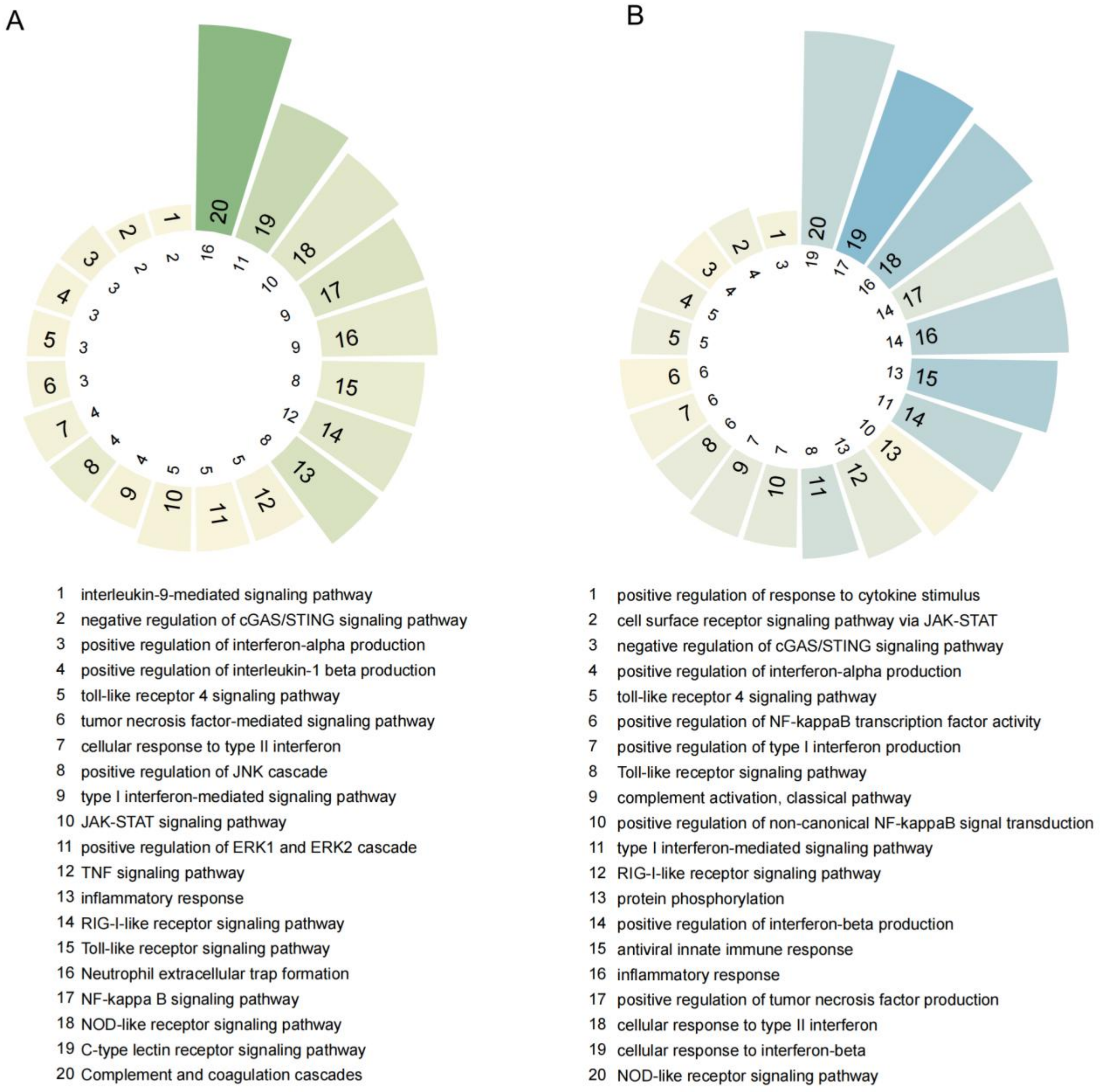

Fig.S4The functional enrichment of those innate immune-related DEPs of CHIKV-infected rhesus monkeys and mice. (A) circular bar plot showing those clustered innate immune signalling pathways of identified innate immune-related DEPs in CHIKV-infected rhesus monkeys, the inner number standing for the gene count clustered by that relevant signalling pathway. (B) circular bar plot showing those clustered innate immune signalling pathways of identified innate immune-related DEPs in CHIKV-infected mice, the inner number standing for the gene count clustered by that relevant signalling pathway.

Figure S5

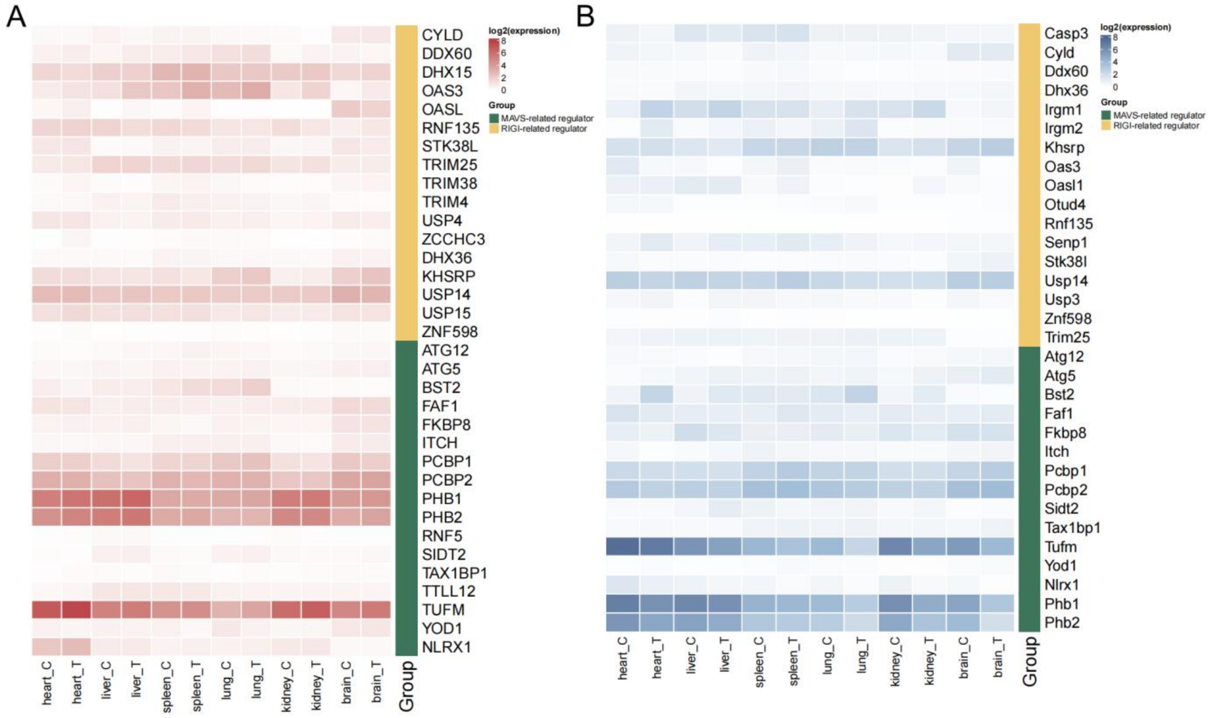

Fig.S5 The expression heatmap of regulators of RIG-I and MAVS in CHIKV-infected rhesus monkeys and mice. (A) heatmap showing the expression of regulators of RIG-I and MAVS in CHIKV-infected rhesus monkeys. (B) heatmap showing the expression of regulators of RIG-I and MAVS in CHIKV-infected mice.

FigureS6

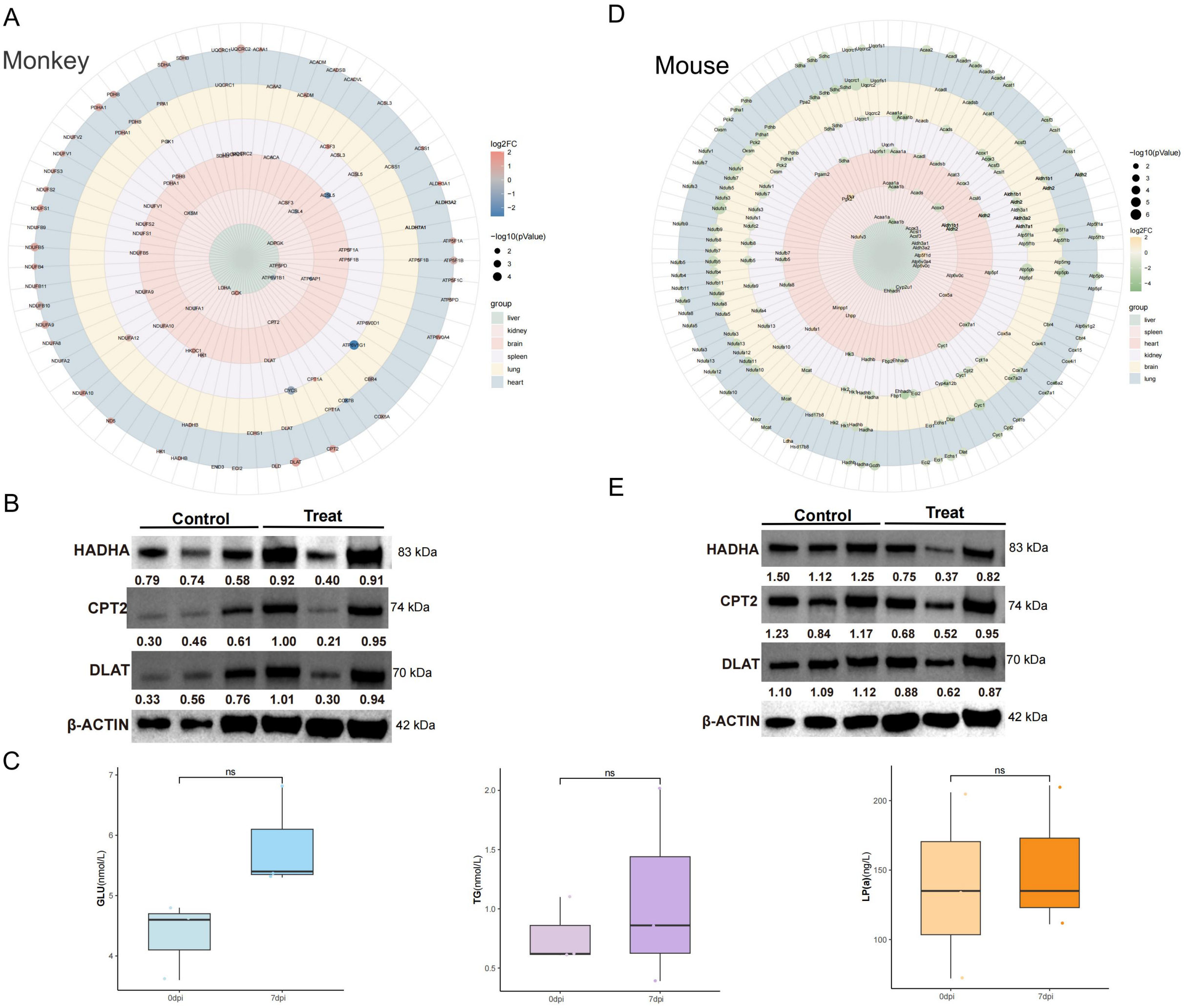

Fig.S6 The metabolic proteomic profiles in organs of CHIKV-infected rhesus monkeys and mice. (A) Circular bubble matrix of immune-related DEPs in all organs of CHIKV-infected rhesus monkeys, with six colours representing each organ. (B) Western-blot results of key proteins in several metabolic pathways verifying the results observed in (A). (C) Box plots showing the expression level of metabolism-related biochemical indicators in CHIKV-infected rhesus monkeys. (D) Circular bubble matrix of immune-related DEPs in all organs of CHIKV-infected mice, with six colours representing each organ. (E) Western-blot results of key proteins in several metabolic pathways verifying the results observed in (D).

Table

Table S1 Analogues of Inarigivir soproxil and their similarity degree

Accession	Compound name	Score
DB15062	Inarigivir	0.946
DB01792	Adenylyl-(3'-5')-uridine 3'-monophosphate	0.917
DB03186	Adenylate-3'-phosphate-[[2'-deoxy-uridine-5'-phosphate]-3'-phosphate]	0.877
DB19132	Renadirsen	0.862
DB03664	P1-(adenosine-5'-P5-(uridine-5')pentaphosphate	0.851
DB01790	(Rp)-cAMPS	0.85
DB03638	Cytidyl-2'-5'-phospho-guanosine	0.844
DB14722	ADU-S100	0.841
DB03280	p1-(5'-adenosyl)p5-(5'-thymidyl)pentaphosphate	0.832
DB18789	Ulevostinag	0.827
DB04514	Uridylyl-2'-5'-phospho-guanosine	0.826
DB02573	2'-deoxycytidine-2'-deoxyadenosine-3',5'-monophosphate	0.825
DB13242	Bucladesine	0.825
DB02527	Cyclic adenosine monophosphate	0.824
DB18889	Dazostinag	0.823
DB03326	Deoxycytidylyl-3',5'-guanosine	0.815
DB02694	Pantoyl Adenylate	0.812
DB04201	Histidyl-Adenosine Monophosphate	0.81
DB01812	Adenosine 3',5'-diphosphate	0.81
DB01895	Aspartyl-Adenosine-5'-Monophosphate	0.809
DB03905	Succinamide-CoA	0.808
DB03230	Adenosine-5'-Propylphosphate	0.807
DB01846	Oxidized coenzyme A	0.804
DB03169	(S)-Hmg-Coa	0.804
DB13046	Tocladesine	0.802
DB01992	Coenzyme A	0.802
DB03117	2-carboxypropyl-coenzyme A	0.8

Table S2 Five Inarigivir soproxil analogues and their molecular docking affinity with RIGI

Accession	Compound name	affinity/(kcal/mol)
DB15063	Inarigivir soproxil	-9.4
DB01792	Adenylyl-(3'-5')-uridine 3'-monophosphate	-9.4
DB03447	Uridylyl-2'-5'-phospho-adenosine	-9
DB03186	Adenylate-3'-phosphate-[[2'-deoxy-uridine-5'-phosphate]-3'-phosphate]	-8.1
DB03664	P1-(adenosine-5'-P5-(uridine-5')pentaphosphate	-8.1
